# Effectiveness and impact of the cross-border healthcare model as implemented by non-governmental organizations: case study of the malaria control programs by health poverty action on the China-Myanmar border

**DOI:** 10.1186/s40249-016-0175-0

**Published:** 2016-09-01

**Authors:** Jun Zhang, Jia-Qiang Dong, Jia-Ying Li, Yue Zhang, Yang-Hui Tian, Xiao-Ying Sun, Guang-Yun Zhang, Qing-Pu Li, Xiao-Yu Xu, Tao Cai

**Affiliations:** 1 Health Poverty Action, Yunnan, People’s Republic of China; 2 Pu’er Center for Disease Control and Prevention, Yunnan, People’s Republic of China

**Keywords:** Malaria, Health poverty action, Cross-border, Non-governmental organization, Yunnan, China, Myanmar

## Abstract

**Background:**

In the Yunnan province of China, 18 counties in six prefectures border Myanmar. Due to its particular combination of geographic features, climate conditions, and cultural landscape, the area provides a suitable environment for the spread of insect-borne diseases such as malaria. In five identified Myanmar Special Regions along the China-Myanmar border, economic development is lagging, people live in extreme poverty, and the healthcare system is fragile. Coupled with political and other reasons, this precludes malaria control work to be effectively carried out in Myanmar, resulting in a heavy burden of the disease. Frequent population movements and favorable conditions for malaria transmission on the border fuel difficulties in controlling and eliminating the spread of the disease in the area.

**Case presentation:**

To reduce the prevalence of malaria in the China-Myanmar border area and improve healthcare services for local residents in this particular environment, Health Poverty Action (HPA) has provided malaria aid in the area since the beginning of 2006, as a sub-recipient of the China Global Fund Malaria Programs. In this case study, we examined HPA’s activities as part of its malaria control programs in the area, analyzed and summarized the effectiveness and impact of the cross-border healthcare model as implemented by non-governmental organizations, and put forward suggestions for cross-border health aid models and for the prevention of malaria transmission in the Greater Mekong Subregion.

**Conclusions:**

HPA had carried out a great quantity of successful malaria control activities in border areas between China and Myanmar, strengthened the partnership and established the collaboration, coordination and cooperation channels among stakeholders. HPA has laid good groundwork and developed its valuable model that could be highlighted and referenced.

**Electronic supplementary material:**

The online version of this article (doi:10.1186/s40249-016-0175-0) contains supplementary material, which is available to authorized users.

## Multilingual abstracts

Please see Additional file 1 for translations of the abstract into five official working languages of the United Nations.

## Background

With a length of 2,186 kilometers, the China-Myanmar border is naturally and geographically susceptible for the existence and spread of a variety of infectious diseases, such as malaria (including multidrug-resistant falciparum malaria) and dengue fever [1–6]. In the five Myanmar Special Regions along the China-Myanmar border, the main source of local income is grain cultivation, with some of the income also coming from rubber and sugarcane cultivation. People live in extreme poverty and education levels are generally low. In these Regions, socioeconomic underdevelopment, an unstable political situation, relatively weak healthcare infrastructure, and difficulties in accessing healthcare services all provide challenges in the implementation and maintenance of effective malaria prevention and control. Consequently, there is a high incidence of malaria and numerous outbreaks. The long China-Myanmar borderline has no natural barriers, which makes it subject to frequent cross-border migration that is difficult to manage through the appropriate channels. Importation and risk of malaria transmission due to the floating population continues to increase.

Founded in 1984, Health Poverty Action (HPA) is an international charitable non-governmental organization (NGO) engaged in healthcare development, with its headquarters in London, United Kingdom. HPA is committed to helping remote, poor, and vulnerable populations access basic healthcare services. Currently, the organization is working in 15 countries across Asia, Africa, and Latin America to prioritize fundamental health services for the most marginalized, remote, conflict-affected, and ignored populations.

HPA established an East-Asian program office in Kunming, Yunnan to manage programs in China and Myanmar. After the signing of the ceasefire agreement between central and local governments of Myanmar in 1994, HPA started work in the Kachin State Special Region 2 and gradually expanded to Shan State Special Region 4, Wa State Special Region 1, Kokang State Special Region, and Kachin State Special Region 1. Cooperating with local health sectors, HPA has implemented programs on primary healthcare, prevention and treatment of malaria and acquired immunodeficiency syndrome (AIDS), and reduction of drug abuse. It has emphasized local capacity-building and sustainable development, and was the first international NGO in the Special Regions of Myanmar to carry out malaria aid programs [7].

In this case study, we examined the activities of the HPA malaria control programs, analyzed and summarized the effectiveness and impact of the cross-border healthcare model implemented by NGOs, and put forward suggestions for cross-border health aid models and for the prevention of malaria transmission in the Great Mekong Subregion (GMS).

## Case presentation

### HPA’s mode of operation and experience

#### Design based on national strategic planning and local needs

In 2010, Ministry of Health (MOH) of the People's Republic of China along with 13 other ministries issued the “Action Plan of China Malaria Elimination (2010–2020),” clearly stating the national goal that all local malaria cases should be eliminated, with the exception of the Yunnan border region, by 2015, and that malaria should be eradicated entirely throughout China by 2020 [8]. In the five Myanmar Special Region communities along the China-Myanmar border, malaria remains severe, and is the main source for imported malaria in Yunnan. Hence, cross-border migration between the two countries directly affects the national goal of eliminating malaria in China. To address this, HPA, the Yunnan Institute of Parasitic Diseases, and the Yunnan Entry-Exit Inspection and Quarantine Bureau, led by the Yunnan Provincial Health Department, jointly applied for funding from the Global Fund to Fight AIDS, Tuberculosis and Malaria (GFATM) Round 10 in 2012, with the aims of strengthening malaria control on the Myanmar border, curbing imported cases, and promoting malaria elimination.

HPA has provided other long-term development assistance for health-related work in the China-Myanmar border area. Compared with other organizations, HPA is more familiar with the health needs and status of health services on the Myanmar side. The organization’s work is an important supplement to the national strategic plan to control malaria and is driven by the aim of improving local malaria control.

### Establishing network management according to the local situation

**Fig. 1:**
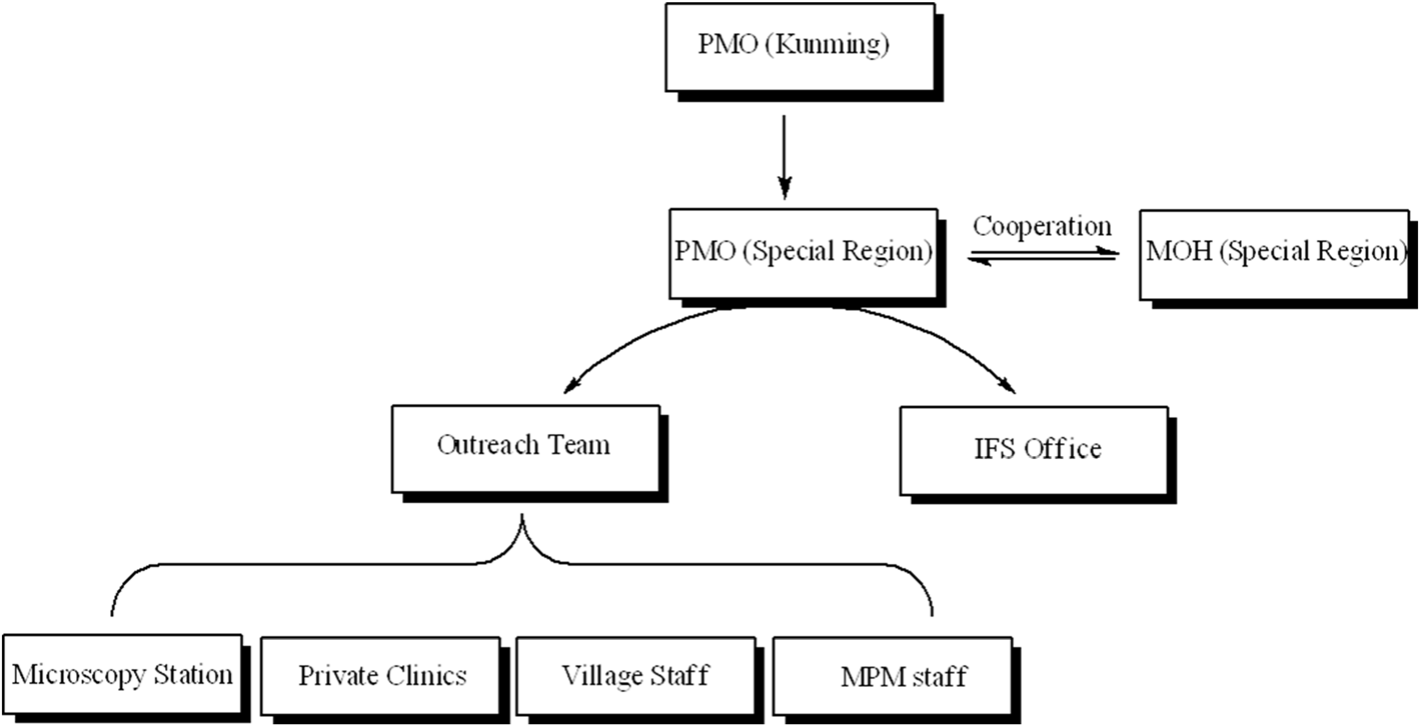
Program management and network structure of HPA activities undertaken as part of the Global Fund initiative on the China-Myanmar border

HPA operates in a realistic manner by closely tailoring program management requirements with local needs and developing program management systems and models that are adapted to local characteristics. Under the management of the Kunming office, five HPA program management offices (PMOs) have been set up with support from local health administrative departments. Each PMO has outreach teams, an information officer, a financial officer, a supply chain management officer, and other program-related officers. To ensure the delivery of services to various regions and target populations, outreach team members not only establish mobile clinics and conduct health education campaigns, but also manage regional microscopy stations, private clinics, village malaria workers, and mobile malaria workers (see Fig. 1). With the long-term support from HPA, a data collecting and reporting system for healthcare networks, including counties, villages, and towns, in a relatively large area across the Myanmar State Special Regions, has been implemented. The system includes primary healthcare data, as well as information on malaria prevention and treatment programs. All basic data can be promptly captured and reported.

### Scientific management and its effective implementation in compliance with program requirements and national technical specifications

According to local conditions, HPA has developed a unified multilingual document for the control and management of cross-border malaria, with standardized criteria, including criteria for business development, financial management, data reporting, monitoring and evaluation, material procurement, and logistics. In terms of human resources, there is a balance between the numbers of Chinese and Myanmar employees, with stability in key positions. By regularly organizing trainings, meetings, and external oversight audits, all program team members can play to their strengths, not only at the local level but also at national policy level. Quality of work is ensured by strictly following program requirements.

HPA attaches great importance to the stability and continuity of its programs. To avoid major changes, misunderstandings, and a loss of trust, a startup meeting is held on a yearly basis, where the work plan and budget are clearly introduced and outlined. Data are collected monthly and used to report on the programs’ progress so that all parties can understand it.

### Strengthening partner management and establishing communication and cooperation channels

Communicating and collaborating with all departments in cross-border programs can be difficult and challenging. HPA has 10 years of accumulated program experience on the China-Myanmar border, and its Myanmar and Chinese units have established a good relationship of trust and cooperation with each other, a key element in the success of the programs.

On the Myanmar side, HPA has always expressed complete neutrality, that is, adherence to the principle of zero involvement in military activities, or politics or religion. Additionally, HPA maintains openness and transparency in the progress of the programs and financial operations, with timely communication with all parties when problems arise. Confronting the new political situation in Myanmar, as well as in the Special Regions, HPA has adjusted its strategy to achieve legal status in Myanmar, including formal registration, signing of a memorandum of understanding, the establishment of liaison offices, and related activities to provide a political guarantee for the continued smooth implementation of the programs.

On the Chinese side, HPA is an important partner in the China-Myanmar malaria control and elimination program, receiving strong support from the Chinese MOH, the Yunnan Provincial Health Department, border states, and county health administrative departments. HPA has also offered considerable support and assistance to China in terms of providing information, personnel, vehicles, and logistical support.

### Respect for local culture and emphasis on local capacity-building for sustainable development

In contrast to some other international organizations, HPA never judges the lack of local technical or cultural development. Based on program experiences and local characteristics, HPA takes action while respecting local religious beliefs, living habits, and the ideas and wishes of residents. When implementing health cooperation programs, HPA not only considers disease issues but also local participation in the programs, aiming to build and strengthen the capacity of local health systems, as well as promote the integration of national health systems [9, 10]. HPA has cooperated with local health departments to fully utilize existing health agencies and personnel. Furthermore, through the establishment of a long-term relationship of trust with the local government, HPA has gradually guided it to invest more in health care. HPA also actively takes responsibility for health development, which is eventually finalized in the form of government policies.

## HPA’s commitment and effectiveness

### HPA’s malaria control programs on the China-Myanmar Border

In 2006, the Chinese MOH and HPA jointly funded and initiated pilot malaria prevention and control program in Kachin State Special Region 2 at the China-Myanmar border area. Through the establishment of an information exchange platform, training of technical personnel, provision of health education to local residents, and the setup of sentinel surveillance, malaria prevention and control on the China-Myanmar border has improved [11–13]. The program has played an important role in the subsequent bilateral cooperation for the prevention and control of infectious diseases in the border area, in addition to institutional and human capacity improvements.

**Fig. 2:**
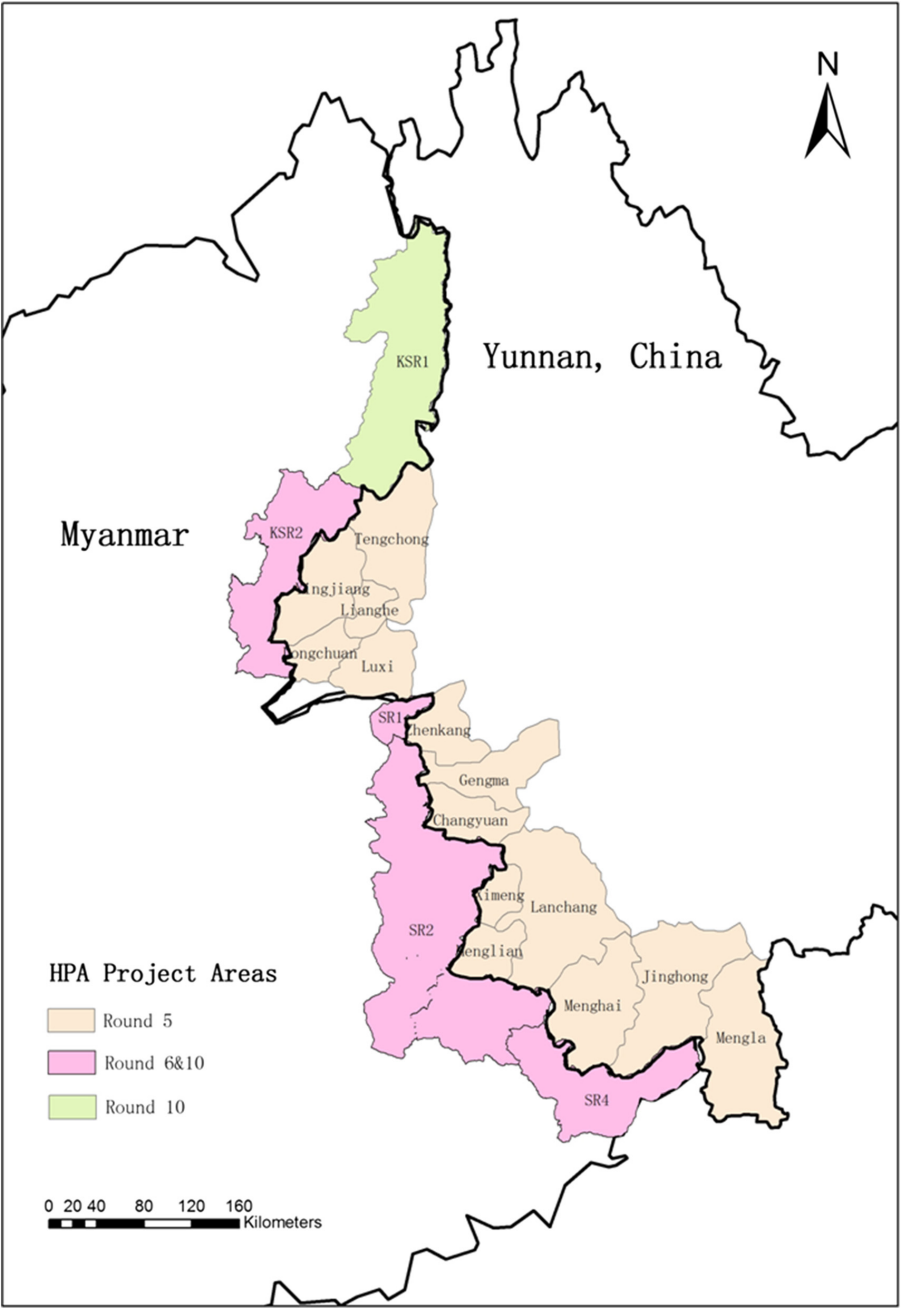
Mapping of regions where HPA’s malaria program is implemented. China Global Fund Malaria Program Round 5 marked in light orange, China Global Fund Malaria Program Round 6 marked in pink, and China Global Fund Malaria Program Round 10 marked in green

Since 2006, HPA has served as a secondary actuator, conducting activities as part of China Global Fund Malaria Program Round 5, 6 and 10. It has successfully carried out malaria prevention and control work in the China-Myanmar border area, which covered 14 counties in Yunnan and five Myanmar State Special Regions (see Fig. 2). After the phase I of China Global Fund Malaria Program Round 10 (January 2012–December 2013), the program has been integrated into Myanmar Global Fund Malaria Program Round 9 (January 2014–December 2016) [14–17].

### Antimalarial services delivered

By the end of 2013, HPA had set up an antimalarial network consisting of 80 malaria diagnosis and treatment stations, 160 microscopic examination technicians, 11 outreach teams, 102 private clinics, 273 village malaria workers, and 42 mobile malaria workers across the five Myanmar Special Regions on the border. It provided blood tests for 587 944 fever patients, treatment for 205 264 malaria patients, and issued 670 353 long-lasting insecticidal-treated nets (LLINs) (see Table 1).

**Table 1:** Antimalarial services of provided by the HPA as part of the Global Fund initiative, 2008–2013

Services/Year	2008	2009	2010	2011	2012	2013	Total
Diagnosis stations	66	66	66	66	66	80	80
Microscopy staff	132	144	160	160	160	160	160
Outreach teams	8	8	8	8	11	11	11
Private clinics	-	-	-	-	80	102	102
Village malaria workers	-	-	-	-	125	273	273
Mobile malaria workers	-	-	-	-	26	42	42
Tests for fever patients	165 669	124 311	71 055	42 726	72 788	111 395	587 944
Malaria treatment	65 027	53 910	26 336	20 840	17 108	22 043	205 264
Distribution of insecticide-treated nets	86 364	123 260	131 408	158 260	104 609	66 452	670 353

### A decreasing malaria burden in the border and changes in knowledge, attitudes, and practices regarding malaria among the population

**Fig. 3:**
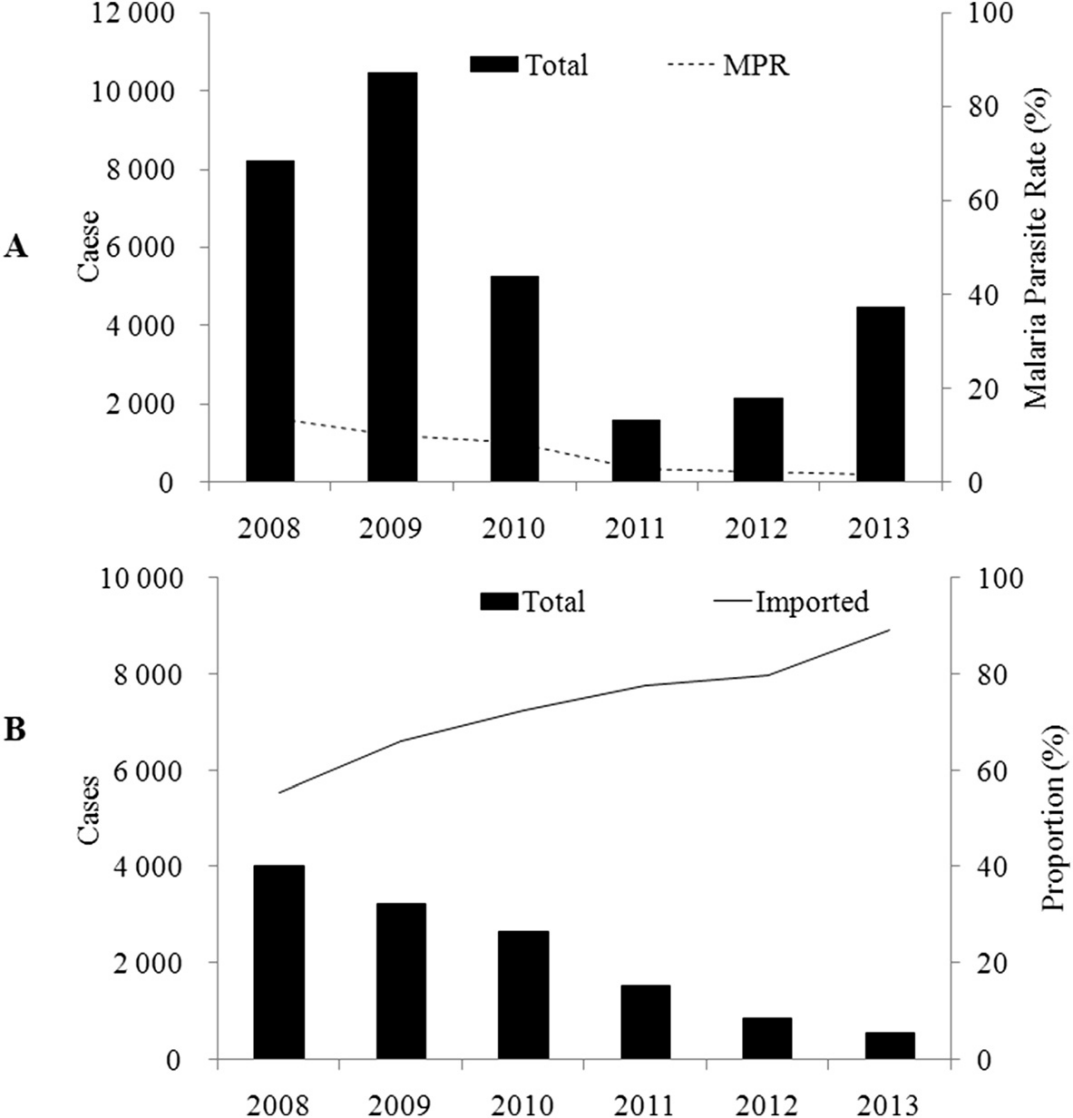
Malaria prevalence in the five Special Regions in Myanmar (**a**) and in the Yunnan province, China (**b**). The black dash line represents the malaria parasite rate in five Special Regions in Myanmar (rate given by HPA). The black solid line represents the proportion of imported cases in Yunnan province (rate given by the web-based reporting system)

Through implementation of the cross-border malaria programs, the malaria parasite rate (MPR) in the five Myanmar areas has declined from a baseline of 13.63 % (2008) to 1.50 % (2013). In addition, the proportion of persons screening positive for falciparum malaria has dropped to 25.70 %. No malaria deaths were reported in 2013, indicating that local malaria prevalence had been effectively reduced (see Fig. 3a). In addition, the proportion of people who had received malaria education among the high-risk population increased from 16.60 % (2007) to 68.44 % (2013). The proportion of local households using LLINs increased from 28.31 % (2008) to 57.30 % (2013), with 77.54 % and 72.76 % of pregnant women and children under five years of age using the nets, respectively.

### Contributions to malaria prevention and control on the Chinese side

From 2006 to 2013, measures implemented on the China-Myanmar border by HPA in partnership with the stakeholders not only reduced malaria prevalence on the Myanmar side but also on the Yunnan province, China side (see Fig. 3b). According to annual Chinese malaria data [18–25], there were 533 malaria cases reported in Yunnan in 2014, including both locally-acquired infections and imported cases, a considerable drop from the 9,770 cases reported in 2007 (a reduction of 94.5 %) when the HPA program had just been initiated. Although the Chinese government has also made a considerable effort to achieve this figure, HPA has undeniably played an extremely positive role in malaria control along the China-Myanmar border, particularly in terms of malaria prevention and control among the floating population.

### Contributions to malaria prevention and control in Myanmar

Alongside progressive economic development and settlement of residents in the Special Regions of Myanmar, local governments have begun to pay attention and attach importance to health problems. They have actively participated in local health development plans and increased investment in health development. This transformation is closely related to the promotion and advocacy led by HPA, which has provided technical support and feasible suggestions for government policy-making that have been recognized by the local government. When phase II of China Global Fund Malaria Program Round 10 was shifted to Myanmar, HPA strictly followed Myanmar national norms. It assisted with incorporating regional malaria information into national malaria information, which the central Myanmar government has begun to extract from local data, thus benefitting the further development and implementation of the national malaria control programs in Myanmar.

### Promotion of health communication and cooperation between China and Myanmar

The HPA cross-border malaria control programs have played a bridging role between the Chinese and Myanmar health sectors, as well as between local and central Myanmar health sectors. HPA has held startup meetings and other communication events, such as countermeasure seminars, and joint advocacy and intervention activities. By inviting tripartite health officials and technical personnel to participate, HPA has promoted communication and cooperation, especially in the area of malaria information reporting, and improved understanding of local demands among stakeholders.

## Potential challenges

### Sustainability of the partnership and working mechanisms

Continued political instability, military conflict, and other issues in the China-Myanmar border area create ongoing challenges to the sustainability of the current program. In addition, as the program has involved various units and partners, communication problems have arisen. Therefore, HPA continues to face challenges in long-term partnership management and effective maintenance of working mechanisms.

### Capacity-building for sustainable development

Although HPA has prioritized local capacity-building throughout the operational area, there remains an imbalance in the implementation and effectiveness of the program between regions. Local staff is recruited from the community based on program requirements. With low subsidies, family responsibilities, and limited personal development, staff turnover is high. HPA should continue to focus on human resource development and malaria control technologies, in order to progressively improve program management.

### Sustainability of funding

Relying on external financial support means that any loss of funding will make local malaria preventive measures difficult to maintain. Even today, due to financial problems, the malaria control program cannot be fully implemented. Bonds between the HPA program and investors must be strengthened, and financing channels should be expanded through the HPA applying for financial support from a variety of funders.

### Strengthening professional and technical support

HPA has been engaged in malaria control programs for many years, with program personnel mainly coming from a medical background; however malaria management remains a highly specialized discipline, especially in the GMS where its epidemiology is relatively complicated. Consequently, professional and technical support for effective malaria prevention and control measures required to eventually eliminate malaria in the region is of great importance. HPA must continue to maintain close cooperation with the China-Myanmar bilateral professional units, assuring diagnostic quality control, implementing standard treatment for patients, and vector control, as well as continuing epidemiological investigations, establishing reporting systems, and improving technical support for mobile population management.

## Implications and recommendations

### Inspiration from HPA’s cross-border malaria control programs

Based on the effective practices of the HPA cross-border malaria control programs, government attention on cross-border malaria and cross-border bilateral cooperation is crucial, which has provided a platform for the design, application, and implementation of cross-border healthcare programs.

As an international NGO working in Myanmar and China, HPA has advantages over other bilateral parties, and has been able to lay good groundwork as well as further develop its model using its valuable accumulated experience. Based on HPA’s model, it is recommended that foreign bodies providing health aid should [26]:

- attach importance to the participation of local governments and capacity-building for local healthcare systems;
- consider the demands and priorities of local recipients;
- be clearly planned and maintain continuity of aid;
- establish a sound management and implementation team, as well as robust management systems and implementation of norms;
- respect local social and cultural characteristics, taking appropriate measures and integrating local resources;
- emphasize cross-national, interagency, and multi-party cross-sectoral coordination and cooperation; and
- play the full role of and enhance communication between NGOs.

### Advice for Chinese health aid programs

#### Increase health investment and promote multilateral aid channels

Chinese foreign investment in healthcare assistance remains limited, with most recipients situated in African and other Asian countries. The proposed “Vision and Actions on Jointly Building Silk Road Economic Belt and 21^st^-Century Maritime Silk Road” from the Chinese government suggests that cooperation on health care with neighboring countries along the “way” needs to be improved [27]. The development of China health aid programs need to be further strengthened in strategic management. This may include gradual improvement to decision-making systems, development of management systems and safeguarding mechanisms, the setting up of an independent foreign aid agency, strengthening of interministerial coordination mechanisms for foreign aid, the establishment of site management agencies, the formation of a scientific evaluation system, the enhancement of human resource management for foreign aid, the boosting of multi-party cooperation, the institution of laws and regulations, and the development of a robust statistical system [28, 29].

Moreover, besides of insistence on the successful model of bilateral programs, in this respect the China-Myanmar cross-border malaria control program has provided valuable lessons to China and Myanmar, further informing future multilateral cooperation and the implementation of disease prevention and control programs.

#### Support NGO participation in foreign aidprograms

The success of the China-Myanmar cross-border malaria program suggests that HPA has strong management and implementation capacity, an example to others in encouraging NGO participation, integrating social resources, and achieving better aid effectiveness. Social academic communities can gain their own advantages by promoting international academic cooperation and communication, sharing information and resources, and sourcing funds. As an example, a high-impact domestic and international public health NGO, the China Preventive Medicine Association has signed a strategic partnership agreement with HPA, and the two organizations have established an NGO platform and network providing strong support for infectious disease prevention and control in the GMS [30].

#### Further strengthen prevention and control of cross-border malaria in the GMS

Malaria in the GMS is a globally high-profile public health concern, particularly because it involves cross-border malaria by a floating population and artemisinin resistance, both of which pose a serious threat to both local and global malaria control and elimination. The Asia Pacific Leaders Malaria Alliance and the World Health Organization has developed respectively its regional strategy for malaria elimination [31, 32]. Additionally, the pragmatic cooperation on malaria has been written by the Chinese government into the national strategic plan of “Vision and Actions on Jointly Building Silk Road Economic Belt and 21^st^-Century Maritime Silk Road”. This has important practical significance and provides a vision for policy-making to further strengthen cross-border cooperation in malaria prevention and control in the GMS, particularly in the China-Myanmar border region.

For future cross-border cooperation in malaria prevention and control in the GMS, governments should continuously lead the work, and bilateral and multilateral cooperation programs should be promoted. The advantages of partnering with an NGO to implement programs in a politically complex area should be valued. The advantages of professional organizations providing technical support and social academic groups in China and Myanmar providing academic information and engaging cooperation with each other should also be highly valued.

## Conclusions

HPA had carried out a great quantity of successful malaria control activities in border areas between China and Myanmar, strengthened the partnership and established the collaboration, coordination and cooperation channels among stakeholders. HPA has laid good groundwork and developed its valuable model that could be highlighted and referenced.

## Abbreviations

GMS, greater mekong subregion; HPA, health poverty action; MPR, malaria parasite rate; LLIN, long-lasting insecticide-treated net; MOH, Ministry of Health; NGO, non-governmental organization; PMO, program management office

## Additional file

Additional file 1: Multilingual abstracts in the five official working languages of the United Nations. (PDF 375 kb)
