# Supplementary material for: Effectiveness and impact of the cross-border healthcare model as implemented by non-governmental organizations: case study of the malaria control programs by health poverty action on the China-Myanmar border
Source: Infect Dis Poverty. 2016 Sep 1;5(1):80. doi: 10.1186/s40249-016-0175-0 (PMC5007861; doi:10.1186/s40249-016-0175-0)
Supplement: Additional file 1: — Multilingual abstracts in the five official working languages of the United Nations. (PDF 375 kb) [file 40249_2016_175_MOESM1_ESM.pdf]

Translation of the abstract into the six official working languages of the United Nations

فعالية وتأثير نماذج الرعاية الصحية عبر الحدود كما تنفذها المنظمات غير الحكومية: دراسة حالة لبرامج مكافحة الملاريا لمؤسسة هيلث بوفرتي أكشن من أجل مكافحة الفقر على الحدود بين الصين وميانمار

يونيو تشانج جيانج تشيانج دونج، جيا يينج لى يو تشانج يانج هوي تيان شياو يينج صن، جوانج يون تشانج تشينج-بو لى شياو يو شو، وتاو كاي

ملخص

خلفية: في مقاطعة يوننان في الصين، 18 محافظة في ست ولايات على الحدود مع ميانمار. وبسبب مزيج أن بها خاص من الميزات الجغرافية والظروف المناخية، والمشهد الثقافي، توفر المنطقة بيئة مناسبة لانتشار الأمراض التي تنقلها الحشرات مثل الملاريا. في خمسة أقاليم في ميانمار تقع على طول الحدود بين الصين وميانمار، التنمية الاقتصادية متخلفة، والناس يعيشون في فقر مدقع، ونظام الرعاية الصحية ضعيف الإمكانات. وبالإضافة إلى أسباب سياسية وغيرها، يحول هذا دون مكافحة الملاريا التي يتعين الاضطلاع بها على نحو فعال في ميانمار، مما أدى إلى عبء ثقيل من المرض. تحركات السكان المتكررة والظروف المواتية لانتقال الملاريا على الحدود تحد من السيطرة والقضاء على انتشار المرض في المنطقة.

حالة العرض: للحد من انتشار الملاريا في منطقة الحدود بين الصين وميانمار وتحسين خدمات الرعاية الصحية للسكان المحليين في هذه البيئة المحددة، قدمت مؤسسة هيلث بوفرتي أكشن (HPA) المساعدات لمكافحة الملاريا في المنطقة منذ بداية عام 2006، كمتفيد فرعي من برامج الصندوق العالمي للملاريا في الصين. في دراسة الحالة هذه، درسنا أنشطة HPA كجزء من برامجها لمكافحة الملاريا في المنطقة، وتحليلها وتلخيصها فعالية وتأثير نموذج الرعاية الصحية عبر الحدود كما تنفذها المنظمات غير الحكومية، ووضع اقتراحات المساعدات الصحية عبر الحدود ونماذج للوقاية من انتقال الملاريا في منطقة الميكونج الكبرى.

الاستنتاجات: قامت HPA بعدد كبير من الأنشطة الناجحة لمكافحة الملاريا في المناطق الحدودية بين الصين وميانمار، وتعزيز الشراكة وتأسيس قنوات التعاون والتنسيق والتعاون بين الجهات المعنية. وقد وضعت HPA الأساس الجيد ووضعت نموذجا قيما يمكن إبرازه والإشارة إليه.

Translated from English version into Arabic by Mahmoud Sami, through

TRANSLATORS
WITHOUT BORDERS

非政府组织跨境卫生援助模式的成效与影响——HPA 中缅边境疟疾援助项目案例分析

Jun Zhang, Jiang-Qiang Dong, Jia-Ying Li, Yue Zhang, Yang-Hui Tian, Xiao-Ying Sun, Guang-Yun Zhang, Qing-Pu Li, Xiao-Yu Xu, and Tao Cai

摘要

引言：中国云南省有 6 个州（市）的 18 个县（市）与缅甸接壤，由于特殊的地理环境、气候条件和人文景观，特别适合疟疾等虫媒传染病的传播和流行。沿云南中缅边境一侧的 5 个缅方特区社会经济发展落后，人民生活极端贫困，卫生服务体系脆弱，加之政治等方面的原因，缅方各种防治工作不能得到有效开展，疟疾负担一直较重。边境人口流动频繁和边境地带良好的疟疾传播条件，是中缅边境地区疟疾流行较其它地区严重并难于控制和消除的重要原因。

案例陈述：鉴于特殊的政治环境，为降低中缅边境地区疟疾流行程度，改善当地居民健康水平，英国无国界卫生组织（Health Poverty Action, 以下简称 HPA）作为中国全球基金疟疾项目的非政府组织次级执行机构，自 2006 年始便在中缅边境地区开展疟疾援助工作。本文以 HPA 中缅边境疟疾援助项目为例，分析和总结了非政府组织跨境卫生援外模式的成效与影响，提出了对今后中国卫生援外模式及大湄公河次区域疟疾跨境防控工作的建议和思考。

结论：HPA 已开展了大量的活动用以中国和缅甸双方的疟疾控制和消除，这大大加强合作伙伴的管理，建

立良好的合作交流和共同协调渠道。HPA 在长期工作中奠定了基础，并积累了有价值的模式经验，其宝贵的经验积累和模式应总结后为其他非政府组织开展类似项目时参考。

Translated from English version into Chinese by Jun Zhang

Efficacité et impact du modèle de soins transfrontalier mis en œuvre par les organisations non gouvernementales : étude de cas des programmes de lutte contre le paludisme de Health Poverty Action à la frontière entre la Chine et le Myanmar

Jun Zhang, Jiang-Qiang Dong, Jia-Ying Li, Yue Zhang, Yang-Hui Tian, Xiao-Ying Sun, Guang-Yun Zhang, Qing-Pu Li, Xiao-Yu Xu et Tao Cai

Résumé

Contexte: Dans la province chinoise du Yunnan, 18 circonscriptions appartenant à 6 préfectures sont frontalières du Myanmar. En raison d'une combinaison particulière de caractéristiques géographiques, de conditions climatiques et de tissu culturel, l'environnement de cette région est propice à la propagation de maladies véhiculées par les insectes, comme le paludisme. Dans cinq « régions spéciales » du Myanmar frontalières de la Chine, le développement économique marque le pas, les populations vivent dans une pauvreté extrême et le système de santé publique est fragile. Pour ces raisons, et à cause aussi de facteurs politiques et autres, la lutte contre le paludisme ne peut pas être menée efficacement au Myanmar et le poids de la maladie est élevé. Les mouvements fréquents de population et les conditions favorables à la transmission du paludisme à la frontière aggravent encore la difficulté de juguler la propagation de la maladie dans la région et de l'y éliminer.
Présentation du cas: Afin de réduire la prévalence du paludisme à la frontière entre la Chine et le Myanmar et d'améliorer les soins de santé pour les populations de cet environnement particulier, l'ONG Health Poverty Action (HPA) apporte une aide à la lutte contre le paludisme dans la région depuis le début de 2006 et bénéficie du soutien des programmes de lutte contre le paludisme du Fonds mondial en Chine. Dans le cadre de cette étude de cas, nous avons examiné les activités de lutte contre le paludisme de HPA dans la région, analysé et résumé l'efficacité et l'impact du modèle de soins transfrontalier mis en œuvre par les ONG et avancé des propositions de modèles transfrontaliers d'aide à la santé et de prévention de la prévention du paludisme dans la sous-région du Grand Mékong.
Conclusions: HPA a réalisé de nombreuses opérations réussies contre le paludisme dans les régions frontalières entre la Chine et le Myanmar, renforcé les partenariats et établi des circuits de collaboration, de coordination et de coopération entre les parties prenantes. L'ONG a ainsi fait un bon travail de terrain et mis au point un modèle précieux, qui pourrait être mis en avant et utilisé comme référence.

Translated from English version into French by Suzanne Assenat, through

TRANSLATORS
WITHOUT BORDERS

Эффективность и воздействие трансграничной модели здравоохранения, осуществляемой неправительственными организациями: Тематическое исследование программ по борьбе с малярией, осуществляемых Организацией по борьбе с болезнями нищеты (Health Poverty Action (HPA)) на границе Китай-Мьянма

Цзюнь Чжан (Jun Zhang), Цзян-Цян Дун (Jiang-Qiang Dong), Цзя-Ин Ли (Jia-Ying Li), Юэ Чжан (Yue Zhang), Ян-Хуэй Тянь (Yang-Hui Tian), Сяо-Ин Сун (Xiao-Ying Sun), Гуан-Юн Чжан (Guang-Yun Zhang), Цин-Пу Ли (Qing-Pu Li), Сяо-Ю Сюй (Xiao-Yu Xu) и Тао Цай (Tao Cai)

Реферат

Предыстория: Район исследования: Китай, провинция Юнань, 18 уездов в шести округах, граничащих с Мьянмой. Вследствие специфического сочетания географических характеристик, климатических условий и культурного ландшафта, район является подходящей средой для распространения переносимых насекомыми болезней, таких как малярия. В выявленных пяти особых регионах Мьянмы, прилегающих к границе Китай-Мьянма, наблюдается отставание экономического развития, крайняя нищета населения и слабая система здравоохранения. Такая ситуация, в сочетании с политическими и другими причинами, препятствует эффективной реализации мер по борьбе с малярией в Мьянме, что приводит к тяжелому бремени болезни. Контроль и прекращение распространения болезни в районе осложняется частыми передвижениями населения и благоприятными условиями для переноса малярии на границе.

Изложение тематического исследования: В целях сокращения распространенности малярии на границе Китай-Мьянма и улучшения услуг здравоохранения для местного населения в данной конкретной среде, Организация по борьбе с болезнями нищеты оказывает помощь в борьбе с малярией в регионе с начала 2006 года в качестве субреципиента программ Глобального фонда по борьбе с малярией в Китае. В данном тематическом исследовании мы изучили деятельность HPA по борьбе с малярией в регионе, проанализировали и подытожили эффективность и воздействие трансграничной модели здравоохранения, осуществляемой неправительственными организациями, а также выдвинули предложения по трансграничным моделям медицинской помощи и предотвращению распространения малярии в субрегионе Большого Меконга.

Выводы: Организация HPA осуществила значительное число успешных мероприятий по борьбе с малярией в пограничных районах между Китаем и Мьянмой, укрепила партнерство и создала каналы сотрудничества, координирования и взаимодействия между участниками программы. Организацией по борьбе с болезнями нищеты заложен хороший фундамент и разработана ценная модель, заслуживающая первоочередного внимания и рекомендации.

Translated from English version into Russian by Tatyana Johnson, through

TRANSLATORS
WITHOUT BORDERS

Eficacia e impacto del modelo de asistencia sanitaria transfronteriza aplicado por organizaciones no gubernamentales: Estudio de caso de los programas de control de la malaria por Acción Sanitaria contra la Pobreza en la frontera entre China y Myanmar

Jun Zhang, Jiang-Qiang Dong, Jia-Ying Li, Yue Zhang, Yang-Hui Tian, Xiao-Ying Sun, Guang-Yun Zhang, Qing-Pu Li, Xiao-Yu Xu, y Tao Cai

Resumen

Antecedentes: En la provincia de Yunnan en China, 18 condados de seis prefecturas limitan con Myanmar. Debido

a la especial combinación de sus características geográficas, condiciones climáticas y paisaje cultural, la zona ofrece un entorno adecuado para la propagación de enfermedades transmitidas por insectos, como la malaria. En cinco Regiones Especiales de Myanmar identificadas a lo largo de la frontera entre China y Myanmar, el desarrollo económico se está retrasando, la gente vive en la pobreza extrema, y el sistema de atención sanitaria es frágil. Junto con motivos políticos y otras razones, esto impide que el trabajo de control de la malaria se lleve a cabo de manera eficaz en Myanmar, lo que resulta en una pesada carga de la enfermedad. Los frecuentes movimientos de la población y las condiciones favorables para la transmisión de la malaria en la frontera exacerban las dificultades para controlar y eliminar la propagación de la enfermedad en la zona.

Presentación del caso: Para reducir la prevalencia de la malaria en la zona fronteriza entre China y Myanmar, y mejorar los servicios de salud para los residentes locales en este entorno particular, Acción Sanitaria contra la Pobreza (HPA, por su sigla en inglés) ha brindado ayuda para combatir la malaria en la zona desde principios de 2006, como un sub recipiente de los Programas del Fondo Mundial para la Malaria de China. En este estudio de caso, se examinaron las actividades de HPA como parte de sus programas de control de la malaria en la zona, analizando y resumiendo la eficacia y el impacto del modelo de asistencia sanitaria transfronteriza aplicado por las organizaciones no gubernamentales, y se presentaron sugerencias de modelos de ayuda sanitaria transfronteriza y para la prevención de la transmisión de la malaria en la subregión del Gran Mekong.

Conclusiones: HPA ha llevado a cabo una gran cantidad de actividades de control de la malaria exitosas en las zonas fronterizas entre China y Myanmar, fortalecido la asociación y establecido canales de colaboración, coordinación y cooperación entre las partes interesadas. HPA ha sentado una buena base y desarrollado su valioso modelo que podría destacarse y citarse como ejemplo.

Translated from English version into Spanish by Susana Rosselli, through

TRANSLATORS
WITHOUT BORDERS
